# Therapeutic strategies to enhance immune response induced by multiple myeloma cells

**DOI:** 10.3389/fimmu.2023.1169541

**Published:** 2023-05-18

**Authors:** Zhaoyun Liu, Chun Yang, Xiaohan Liu, Xintong Xu, Xianghong Zhao, Rong Fu

**Affiliations:** Department of Hematology, Tianjin Medical University General Hospital, Tianjin, China

**Keywords:** multiple myeloma, immunotherapy, immunogenic cell death, vaccine, neoantigen

## Abstract

Multiple myeloma (MM)as a haematological malignancy is still incurable. In addition to the presence of somatic genetic mutations in myeloma patients, the presence of immunosuppressive microenvironment greatly affects the outcome of treatment. Although the discovery of immunotherapy makes it possible to break the risk of high toxicity and side effects of traditional chemotherapeutic drugs, there are still obstacles of ineffective treatment or disease recurrence. In this review, we discuss therapeutic strategies to further enhance the specific anti-tumor immune response by activating the immunogenicity of MM cells themselves. New ideas for future myeloma therapeutic approaches are provided.

## 1 Introduction

Multiple myeloma (MM) is a hematological malignancy due to a malignant clone of plasma cells and is highly heterogeneous, varying widely in clinical presentation, treatment options and prognosis. Myeloma is incurable and eventually relapses as the disease progresses (1). Over the past three decades there have been huge advances in the treatment of patients with MM. The advent of immunologic agents has revolutionized the treatment paradigm for patients while improving survival rates, with incredible results in both NDMM and relapsed refractory MM(RRMM) patients (2). Immunomodulators(IMIDs)-based therapy has become an essential ingredient in the treatment regimen for patients who are eligible or ineligible for transplantation (3). Monoclonal antibody(mAb) and bispecific antibodies and bispecific therapeutic engagers (BiTEs) therapy harness specific targets on the surface of myeloma cells to trigger an immune response, inducing disease remission. Immune checkpoint inhibitors (ICIs) therapy has shown outstanding performance in combination therapy. The emergence of CAR-T therapy in recent years has resulted numbers of clinical trials being conducted due to rapid onset of action and high response rates, but it cannot be denied the problems of high price and poor treatment persistence (4).

The key to the effectiveness of some immune agents is the expression of specific antigens on the surface of tumor cells (2). The inability to successfully activate antitumor immune responses due to antigen loss is a direct cause of the failure of these conventional immune agents. And how to sufficiently activate the specific anti-tumor immune response is exactly what we have listed in this review.

Generally, immunotherapies have an impact on tumor-host interactions, and effective treatment tilts the balance towards activating an immune response against malignant cells. The “resetting” effect of chemotherapeutic drugs on the immune system and the restoration of immune surveillance are discussed in the review by Zitvogel et al. (5) That successful antineoplastic drugs elicit therapeutic immune responses can be partly explained by enhancing malignant cells sensitivity to immune effect, increasing their antigenicity and immunogenicity (5). That is to say, the core of the therapeutic strategy proposed is to target the tumor cells themselves to increase their ability to elicit an effective immune response, rather than focusing on enhancing immune effector cells as traditional immunological agent model. But this therapy has not been discussed in the context of myeloma.

Here, we discussed immunotherapy strategies to enhance immune response based on MM cells. Immunogenic cell death (ICD) inducers target intact myeloma cells to improve their immunogenicity and achieving immunogenicity death of MM cells. Vaccines based on myeloma cell antigenic peptides or designed to directly load whole myeloma cells realize the purpose of actively releasing self-antigen. The advent of technology to detect neoantigens of myeloma has broken the immune tolerance dilemma and increased the autoantigenicity of MM cells, making them easier to be recognized by the immune system. Boosting signals from co-stimulatory molecules on the surface of myeloma cells appears to make them more sensitive to immune attack.

In this review, we outline the immunodeficiency in myeloma patients and main strategies currently available for immunotherapy. And these therapeutic approaches that target myeloma cells and up-regulate the body’s immune response are summarized.

## 2 Immunodeficiency in multiple myeloma

It is now believed that the development of myeloma is the result of two simultaneous factors (6). One factor is the clonal evolution of tumor cells due to genetic mutations in myeloma patients. Another factor is the alteration in the composition and function of the immune system, leading to vandalized immune homeostasis as well as loss of normal immune surveillance function and formation of immunosuppressive microenvironment. Abnormalities in the immune machinery of myeloma involve many aspects, including immune effector cells deactivation, production of cytokines that promote tumor growth and form an immunosuppressive microenvironment, and the accumulation of myeloid-derived suppressor cells (MDSC) and tumor-associated macrophage (TAM) (7–9).

The specific mechanisms of immune dysregulation in myeloma patients are complex. T cells have numerical and functional defects and aberrant CD4/CD8 T cells ratios. Myeloma cells express programmed cell death ligand 1(PD-L1) and CD155 ligand, which directly lead to T cell depletion by binding to the T cell surface receptors programmed cell death protein 1(PD-1) and T cell immunoglobulin and ITIM domain(TIGIT), respectively (10). Transforming growth factor-β(TGF-β), secreted by MM cells, regulatory T cells(Tregs) and bone marrow mesenchymal stem cells (BMSCs), facilitates the differentiation and expansion of Tregs, which inhibit T-cell function. TGF-β can cause defects in the natural killer cells(NKs) number and function (11). MM cells expressing PD-L1 are also able to suppress NK cytotoxicity (12). Peripheral dendritic cells(DCs) exhibit an immature phenotype and impaired antigen presentation in MM, possibly associated with low expression of co-stimulatory molecules (13). TGF-β and IL-10 secreted from myeloma cells mediate the defective DC function, which could be restored by IL-12 and interferon-gamma (IFN-γ) (14).Indoleamine 2,3-dioxygenase(IDO) is produced by immature DCs and can deactivate T cells (15).

MDSCs and TAMs co-exist in the immunosuppressive microenvironment of myeloma. MDSCs are immature myeloid cells showing heterogeneity. The differentiation of MDSCs in myeloma is blocked by vascular endothelial growth factor(VEGF), granulocyte-macrophage colony-stimulating factor (GM-CSF) and IL-6 (16). Immature MDSCs accumulate in the microenvironment, inhibiting the cytotoxic effects of T cells by arginase, reactive oxygen (ROS) as well as nitric oxide and contributing to cancer progression and metastasis (16, 17). Macrophages can be divided into two subgroups according to their functions, i.e., M1 and M2 (18). M1 macrophages exert anti-tumor effects and act as APCs to activate cellular immune responses against MM antigens. Chemokine (C-X-C motif) ligand 12(CXCL12), chemokines ligand 2(CCL2), chemokines ligand 3(CCL3) and chemokines ligand 14(CCL14) produced by myeloma cells and BMSCs promote macrophage M2 polarization and imbalance the M1/M2 ratio (19, 20). M2 macrophages secrete or release cytokines that promote tumor neovascularization through direct or indirect action (21).

## 3 Current immunotherapy strategies for multiple myeloma

**Figure 1:**
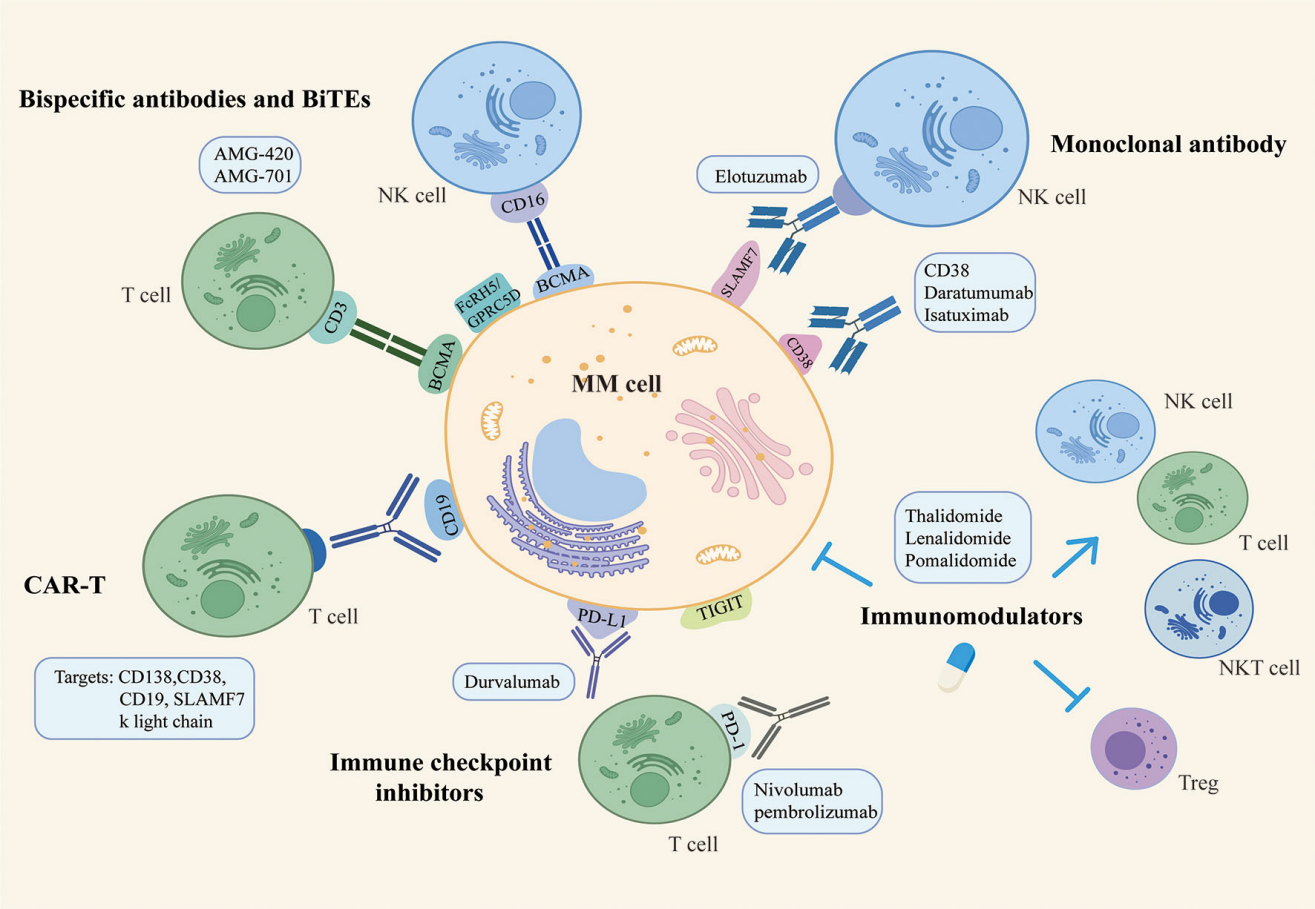
The general immunotherapy strategy for multiple myeloma.

A variety of different immunotherapy strategies for MM are emerging, aiming to increase the depth and breadth of treatment while reducing the incidence of side effects. In recent years, the standard of care for myeloma has been further rewritten with advances in immunotherapy. We currently believe that myeloma immunotherapy focuses on two main areas. On the one hand, membrane surface molecules are used to build a bridge between tumor cells and effector cells, i.e., to provide a peptide chain or antibody to target and link myeloma cells and immune cells, narrowing the gap between them and achieving the specific targeting and killing effect. Examples include mAbs therapy, BiTEs therapy and CAR-T therapy. On the other hand, considering the existence of immunosuppressive microenvironment and immunosuppressed state in the patient’s body, immune cells frequently fail to perform their normal capacity of recognizing and killing tumor cells. ICIs and IMIDs can restore the depleted state of immune cells and strengthen the function of autoimmune cells. The following summarizes the general immunotherapy strategy for myeloma. (**Figure 1**)

### 3.1 Monoclonal antibody

Immunotherapy with mAb is clinically effective and can benefit even patients with advanced disease stages (22). Food and drug administration(FDA) has approved daratumumab and isatuximab targeting CD38 and elotuzumab targeting SLAM7 for the treatment of myeloma (23). Elotuzumab is an anti- signaling lymphocytes activating molecule factor 7(SLAMF7) mAb that exerts NK cell-mediated ADCC effects or directly activates NK cells to kill tumor cells (24, 25). The mechanism of Dara is more extensive. In addition to directly inducing apoptosis, macrophage-mediated phagocytosis and FC-dependent immune regulation, Daratumumab can also promote the activation and expansion of NK and T cells, restoring their anti-tumor ability (26, 27). Daratumumab has demonstrated outstanding clinical efficacy in myeloma patients, showing fast, in-depth and lasting responses even when administered as a single agent in relapse and refractory MM(RRMM) patients (28). Lenalidomide upregulated the expression of CD38 on the surface of myeloma cells (29), and the combination of Daratumumab with lenalidomide/dexamethasone (30) or other drugs such as bortezomib/dexamethasone are continuously carried out and they can significantly improve the clinical outcomes of patients (29).

### 3.2 Bispecific antibodies and bispecific therapeutic engagers

Bispecific antibodies and BiTEs are emerging immunotherapeutic strategies that are thought to be somehow potentially superior to monoclonal antibodies. They have two binding sites, one end binds to tumor cell surface antigens, currently involved in B cell maturation antigen(BCMA), CD38, CD19, G protein-coupled receptor class C group 5 member D(GPRC5D) and Fc receptor-like 5(FCRL5), and the other end binds to molecules on immune effector cells, such as CD3 on T cell and CD16 on NK cell (31). In other words, the interaction between tumor cells and immune effector cells is no longer dependent on T cell receptor(TCR)-specific recognition and antigen presentation, but directly kills tumor cells while activating immune effector cells (32, 33). BiTEs refers to a structure consisting of single-chain variable fragment(scFV)-binding regions and a short linker, with lower molecular weight and relatively short half-life, thus requiring continuous infusion to maintain the desired therapeutic concentration (34). AMG420 is the first BiTE with confirmed clinical efficacy, targeting both CD3 and BCMA to induce TCR-independent immune response activation and tumor cell death (35). AMG-701 was developed to address the short half-life involved with AMG-420 (36). Anti-FcrH5/CD3 bispecific antibodies are capable of stimulating the formation of immune synapses (37). And the GPRC5D/CD3 bispecific antibodies constructed based on GPRC5D overexpression on myeloma cells can effectively recruit T cells to attack tumor cells (38). Moreover, Elranatamab was granted Orphan Drug Designation by the FDA as a humanized anti-BCMA/CD3 bispecific IgG2a antibody that has demonstrated a relatively high safety and durability of anti-myeloma effects (39). Talquetamab targets both MM cell surface GPRC5D and T cell surface CD3 to lyse target cells while activating cytotoxic T cells. Talquetamab has been approved by the FDA for the treatment of adult RRMM patients who have received at least 4 prior lines of therapy, including proteasome inhibitors, immunomodulators, and anti-CD38 antibodies, due to its significant clinical efficacy (40). Clinical and preclinical studies on the application of bispecific antibodies and BiTEs are being conducted and have shown potential clinical application value.

### 3.3 CAR-T

CAR is a chimeric protein that binding mAb-derived scFv to the T-cell receptor domain and co-stimulatory molecular signalling domain to enhance the T-cell immune response by mimicking T-cell activation *in vivo* (41). These patient-derived T cells are expanded and modified to selectively target tumor antigens and BCMA is most frequently designed for use with CART therapy owing to its high selectivity of expression on MM cells. Other targets include CD138, CD38, CD19, SLAMF7 and k light chain (42). Not only does CAR therapy recognize tumor antigens in a MHC-independent manner and reduced off-target effects, it also has a stronger affinity for antibody-antigen binding and a more rapid onset of action (4). The FDA has approved idecabtagene vicleucel (Abecma) and ciltacabtagene autoleucel (Carvykti) for the treatment of patients with heavily pretreated RRMM due to their high clinical response rate and MRD negativity (43). However, the sustainability of CAR-T treatment is problematic and the risk of relapse after treatment is significant. In addition, loss of myeloma cell surface antigens or relapsed disease that do not express the original CAR target are correlated with treatment ineffectiveness (44). The application of CAR-T also requires consideration of clinical toxicity, most commonly cytokine release syndrome and neurotoxicity due to the rapid expansion and activation of CAR-T cells (45).

### 3.4 Immunomodulators

IMIDs, which include thalidomide, lenalidomide and pomalidomide, have a significant role in improving the prognosis of patients with myeloma and are an essential component of existing treatment regimens. Research now generally agrees that IMIDs have both tumor and immune dual targeting effects (46).IMiDs have directed cytotoxic effects on myeloma cells, inducing growth arrest and apoptosis of MM cells, which is associated with downregulation of interferon regulatory factor 4(IRF4) and cereblon-dependent degradation of the transcription factors Ikaros/Aiolos (IKZF1/3) (47, 48). In terms of immunomodulation, IMiDs promote the immune system and improve the immunosuppressive microenvironment of myeloma by eliminating the adhesion between myeloma cells and BM, upregulating T, NK and NKT cells with the downregulation of Treg, as well as inhibiting angiogenesis (49).Additionally, IMiDs regulate the production of cytokines. Lenalidomide promotes the cytotoxicity and antibody-dependent cell-medicated cytotoxicity(ADCC) of NK and NKT cells by inducing IKZF1/3 degradation and IL-2 secretion in T cells (50, 51).Given that some patients remain sensitive to IMiDs even after relapse, combined IMiDs are also used as a preferred option for patients with RRMM (33). Pomalidomide was approved by the FDA in 2013 for refractory patients who had been received at least two therapeutic regimens, including bortezomib and lenalidomide (52).

### 3.5 Immune checkpoint inhibitors

PD1/PD-L1 are major immune checkpoints, and as negative regulatory axes of immune modulation, their overexpression would lead to immune escape and the formation of immune tolerance in myeloma cells (53). A remarkable increase in PD-L1 expression can be detected in RRMM patients (54). Another negatively regulated immune checkpoint is TIGIT, which inhibits the killing function of NK cells and the cytotoxic effect of CD8+ T cells, affecting antigen presentation and the release of anti-inflammatory factors (55). ICIs are specific mAbs that blocking PD-1/PD-L2 binding and acting by targeting immunosuppressive signals in the tumor microenvironment. However, monotherapy with ICIs has not seen remarkable benefits in the early stages. The PD-1 inhibitors pembrolizumab and nivolumab were not observed objective responses or significant clinical efficacy when used separately in patients with MM (56). These studies highlight that ICIs need to be used in combination with other therapeutic strategies to achieve translation of clinical outcomes. Based on the theoretical basis that Len in combination with ICI is capable of restoring the cytotoxic effects of depleted NK cells and preventing immune evasion by MM cells (57), the combination of ICIs and IMiDs has attracted many investigators. For example, pembrolizumab in combination with lenalidomide and dexamethasone for NDMM, or pembrolizumab in combination with pomalidomide and dexamethasone for RRMM (discontinued due to safety concerns) (58, 59). Furthermore, the strategy of combining multiple ICIs against diverse targets has a potentially synergistic therapeutic effect. In conclusion, ICIs are a viable treatment strategy for patients with MM, but are still being explored and a balance still needs to be found between immune efficacy and pharmacological toxicity.

## 4 Therapeutic strategies to enhance immune response induced by multiple myeloma cells

The effects of immunological agents such as mABs, BITEs and CAR-T, which are now widely used in clinical practice, are dependent on the expression of specific antigens on myeloma cells. However, it is difficult to accurately and consistently monitor the expression of surface antigens in practical treatment. With the progress of treatment, the number of cell clones that do not express specific antigen will gradually increase, and the emergence of immune escape eventually lead to drug resistance. Patients have to switch to other drugs or take the next-line therapy. Therefore, we propose immunotherapeutic strategies that target myeloma cells with a view to circumventing the effects of antigen loss on immunotherapy efficacy.

**Figure 2:**
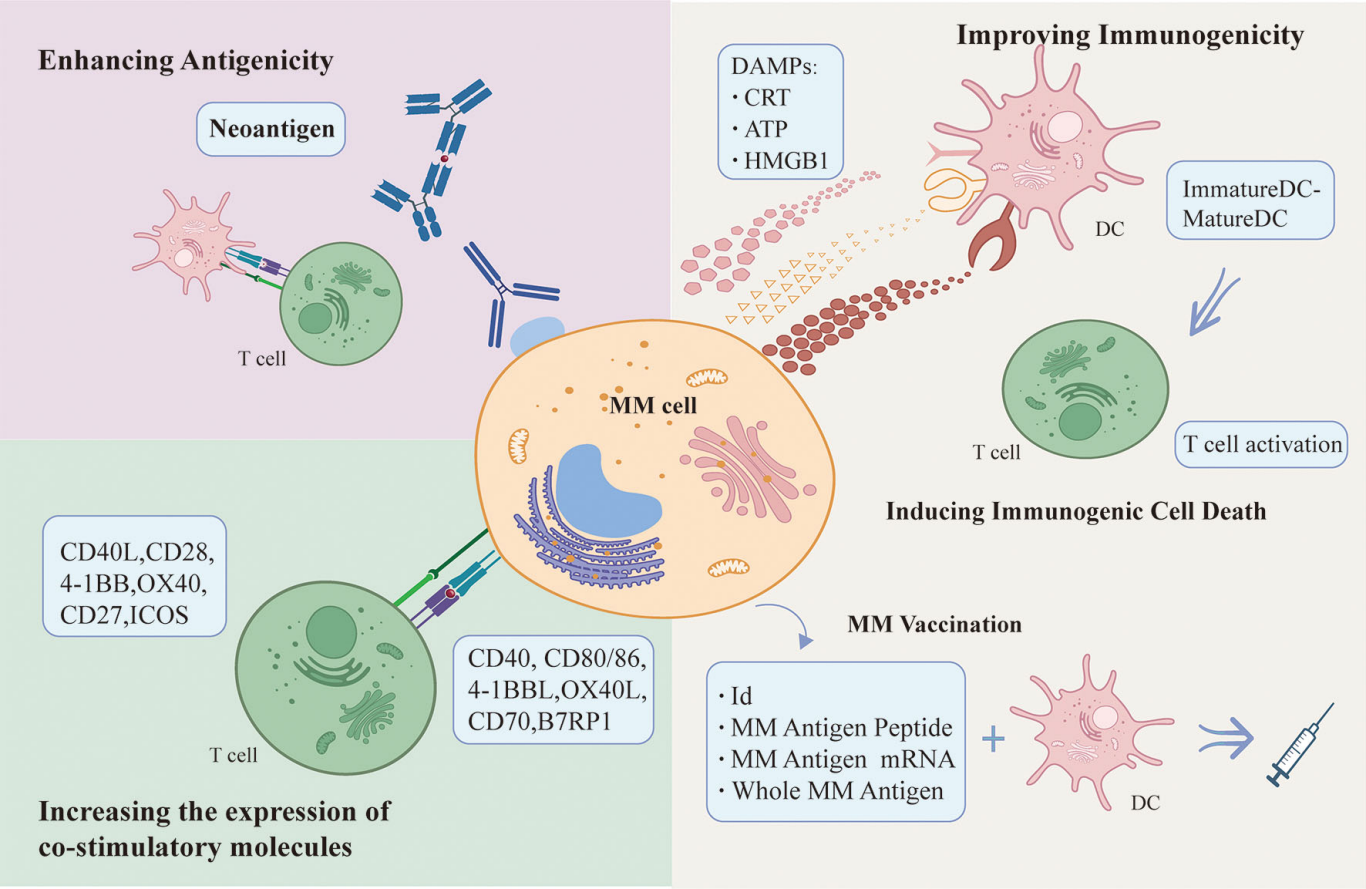
Therapeutic strategies to enhance immune response induced by MM cells.

Immunogenicity is an intrinsic property of antigen that reflects its ability to elicit an immune response. Myeloma cells can provoke some degree of immune response, however, most of the time immune cells are failing to kill tumor cells directly with the occurrence of malignant clones of tumor cells and cancer progression. Both ICD and vaccine treatment strategies aim to stimulate endogenous immune responses against malignant myeloma cells. The efficacy of immunotherapeutic agents would be enhanced by targeting tumor cells to increase their sensitivity to death and improve poorly immunogenic cells recognized by APCs, i.e. targeting ICD of tumors cells. Vaccine formulations can activate immune effector cells to continuously attack tumor cells, benefitting some patients with refractory disease and minimal residual disease (MRD). The identification of neoantigens by new technologies breaks the dilemma of immune tolerance of traditional chemotherapy and fills the gap of ICD and vaccine therapy. Therapeutic approaches that increase the expression of co-stimulatory molecules on the surface of myeloma cells complement the therapeutic strategies that target whole myeloma cells, allowing tumor cells to be successfully presented to DCs as APCs and activate effector T cells. Therapeutic strategies to enhance immune response induced by MM cells were summarized in **Figure 2**.

### 4.1 Inducing ICD formation

The occurrence of ICD is dependent on sequential and concerted release of DAMPs, including the exposure of the calreticulin (CRT) and heat shock protein (HSP), the secretion of adenosine triphosphate (ATP), the release of the non-histone chromatin binding protein high mobility group box 1 (HMGB1) and cytokines such as type I interferon (INF I). DAMPs binding to specific pattern recognition receptors (PRRs) expressed by DCs recruits more APCs and activates their ability to process antigens. In the presence of activating co-stimulatory signals, T cells are successfully activated and immune responses are initiated. However, in the course of the ICD, innate immune responses are simultaneously initiated (60, 61).

Improved immunogenicity: Inducers acts on MM cells to increase MM cells immunogenicity and induce the release of DAMPs from dying MM cells, which bind to PRRs on DCs and initiate T cell activation and anti-tumor immune response. MM-derived DC vaccine can effectively activate immune effector cells to continuously attack MM cells and effectively initiate endogenous immune responses. Enhancing antigenicity: Neoantigens are promising in breaking the drug tolerance dilemma, as they are effectively recognized and killed by T cells, making them a good potential therapeutic target for immunotherapy. Up-regulating the expression of costimulatory molecules such as CD40, CD80/86, 4-1BBL, OX40L, CD70 and B7RP on the surface of myeloma cells can increase the susceptibility of myeloma cells to attack by immune effector cells.

ICD is a type of pro-inflammatory cell death, and the key factors are the generation of sustained reactive oxygen species (ROS) and endoplasmic reticulum stress (ERS). ER is associated with protein synthesis and undergoes an unfolded protein response (UPR), a homeostatic mechanism designed to rapidly detect and correct protein processing errors. When ICD occurs, over-activation of the UPR leads to ERS accompanied by the release of DAMPs to elicit immune responses and the development of memory immunity (62).

After the induction of ICD, dying cells expose and/or release DAMPs. What happens in the first is the transfer of CRT, which acts as an “eat me” signal, from the ER lumen to the cytoplasmic surface. Ecto-CRT exposure accompanied by co-translocation of ERp57 and ERS plays a significant role in it (63). Ecto-CRT interacts with the CD91 receptor on APC to enhance immunogenic recognition and phagocytosis of dead cells (64, 65). Dying tumor cells secrete ATP extracellularly via the autophagic pathway, bind to ionotropic (P2X7) and metabotropic (P2Y2) purinoceptors on the surface of APCs and acts as a “find me” signal to facilitate APCs recruitment (66). Extracellular ATP activates caspase 1-dependent NOD-like receptor thermal protein domain associated protein 3(NLRP3) inflammasome and secretes IL-1b for immunostimulatory effects (67). HMGB1 secreted by tumor cells binds to various PRRs (e.g., Toll-like receptor 4 (TLR4)) and promotes the release of cytokines such as IL-1b. Both the knockdown of HMGB1 and the application of TLR4-neutralising antibodies will deplete the efficacy of the immune response induced by the anthracyclines or cyclophosphamide *in vivo* models (68). Tumor cells undergoing ICD produce IFNI via RNA activation of the TLR3 or cyclic guanosine monophosphate-adenosine monophosphate synthase/stimulator of interferon genes (cGAS/STING) pathways. The IFNI response triggers the release of CXC-chemokine ligand 10 (CXCL10) when it binds to the interferon receptor (IFNAR I) on tumor cells, which exerts an immunostimulatory effect (69, 70).

Currently, there are type I and type II inducers. Type I inducer induces non-ER-targeted apoptosis, causing mild ERS, including anthracyclines, oxaliplatin, bortezomib, cyclophosphamide and radiation. Type II inducers selectively target ER and cause ROS-based ERS. Chrysin photodynamic therapy and lysovirus therapy, etc. are type II ICD inducers (60). Induction of ICDs is a very effective immunotherapeutic approach for targeting myeloma cells in MM as it enhances their immunogenicity and elicits a strong anti-tumor immune effect.

Bortezomib, a proteasome inhibitor (PI), is one of the representative drugs and it has been approved for first-line treatment of MM (71). There are a large amount of monoclonal proteins secreted by MM cells in the peripheral blood. Whereas protein overload in MM cells makes them dependent on proteasome activity. As a PI, bortezomib leads to accumulation of misfolded proteins and produces ROS, inducing immunogenic death of myeloma cells (72). It has been demonstrated that bortezomib induces immunogenicity in myeloma cells by activating the cGAS/STING pathway and producing type I interferon (73). Furthermore, the combination of bortezomib and STING agonists appears to induce a more intense ICD, providing a preclinical foundation for the combination of the two agents to improve the prognosis of MM patients (73). Bortezomib in combination with dexamethasone is currently the standard regimen for RRMM. Clinical trials combining mAbs (74), or a selection of different IMDs (75), are ongoing and some of them have shown excellent efficacy.

The second-generation PI carfilzomib is considered to be more effective and safer than bortezomib. Carfilzomib inhibits proteasome activity in an irreversible manner and misfolded proteins accumulate in the body, leading to significant ERS. A phase 2 study evaluated the efficacy of carfilzomib injection, involving 266 RRMM patients who had received median five prior treatments and mostly were intolerant to bortezomib and lenalidomide (76). ASPIRE (NCT01080391) study showed that combining carfilzomib with Lenalidomide and dexamethasone demonstrated the higher rates of overall response and improved PFS and 2-year overall survival (77). In a phase 3 multicenter randomized controlled study comparing the efficacy of carfilzomib and dexamethasone with bortezomib and dexamethasone for RRMM, the carfilzomib group showed longer progression-free survival(PFS), improved objective response rate(ORR), complete response (CR), very good partial response(VGPR), and median survival time with better clinical advantages than the bortezomib group (78).

Studies have demonstrated that irradiation, hyperthermia (HT), high hydrostatic pressure (HHP), bortezomib and lenalidomide as ICD inducers enhance the immunogenicity of dead tumor cells and augment anti-tumor T cell responses *in vivo* by improving DC function (79). Bortezomib induces HSP90 exposure on the surface of dying cells, which mediates intercellular contact between DCs and dying cells, delivers DC activation signals and successfully activates T cells to kill tumor cells. This targeting of tumor cells and enhancement of their immunogenicity by bortezomib, providing a unique immune activating stimulus has been demonstrated by Spisek et al. (80) Pomalidomide and lenalidomide have been proven to be common ICD inducers in MM. These immunomodulators enhance the uptake and presentation of tumor antigens by DCs and may play an adjuvant role in vaccine therapy (79, 81). In addition, experimental validation of the combination of the DNA methyltransferase inhibitor decitabine and the histone deacetylase inhibitor quisinostat acting on the murine immunocompetent 5T33MM model, vaccination of mice with epigenetic compounds effectively delayed the progression of tumors *in vivo*, activated DC cells and strengthened anti-tumor immunity in the form of inducing immunogenic death of myeloma cells (82). Mechanistically, epigenetic modulators can increase MM cell surface ectocalreticulin, reduce CD47 and PD-L1 expression, promote DC cell maturation and the persistence of immune effector CD4/8 cells (82).

ICD offers an approach to treat MM through combining immunogenic death of tumor cells with the initiation of specific anti-tumor immune responses. ICD makes it possible to achieve long-term anti-tumor effects by breaking down the immune tolerance and immunosuppressive microenvironment in patients. It is now widely accepted that ICD can transform dying tumor cells into a ‘vaccine’ that is able to induce anti-cancer immunity without the addition of any adjuvant (83). However, some questions remain to be addressed. For example, how to translate the results of *in vitro* studies into clinical efficacy (84). In addition, understanding the molecular mechanisms underlying inducers would be useful in transforming non-immunogenic cell death inducers into inducers that effectively induce immunogenicity in tumor cells and will guide the combination of drugs (83).

### 4.2 MM vaccination

MM is featured by malignant clonal proliferation of the plasma cells and the secretion of idiotype antigens (Id), which is monoclonal immunoglobulins (Ig) or fragments (i.e., M proteins). Id has unique amino acid sequences in their variable regions, making them different from normal Ig and can be used as antigens for vaccination. Many researchers have conducted clinical trials of Id vaccines for MM therapy. Id is normally coupled with keyhole limpet hemocyanin (KLH) and supplemented with an immunostimulatory adjuvant such as GM-CSF for simultaneous injection. In some patients, researchers observed Id-specific immune responses, which do not always occur and are short-lived. Clinical responses are rarely observed in a small number of patients as well (85–88). These observations, however, disclose the low immunogenicity of Id and the efficacy needs to be improved.

#### 4.2.1 DC-vaccine

The activation of initial T cells is more dependent on the presence of DC stimulatory signals, making DC the only dedicated antigen presenting cell (APC) that can directly activate initial T cells. As the most powerful APCs, DCs link innate and adaptive immune responses (89). However, MM patients have a quantitative and functional defect in DCs, affected by cytokines such as TGF-β and IL-10 (90). After antigen stimulation, DCs are unable to upregulate surface activating costimulatory molecules, thereby disrupting antigen presentation (91).

MM vaccines based on DCs are undergoing continuous advancements. The source of DCs, the method for maturation induction of DCs, the type of tumor antigens co-loaded with DCs and the technology, and the route of administration will have an impact on the clinical efficacy. Currently, the commonly used DCs are derived from *in vitro* preparations of circulating blood mononuclear DC (moDCs) or bone marrow progenitor cells (92). Maturation and activation of DCs are either accomplished through direct contact with antigens *in vivo* or mimicked *in vitro* by co-culture with cytokines such as prostaglandin E2, pathogen recognition receptor (PRR) agonists or TNF-a. DCs of allogeneic origin compensate for the DC number and defect in function due to autologous origin but may be limited by MHC molecules.

Many clinical studies have included post-HSCT patients in their study populations (93). Post-transplant vaccination may be effective in controlling residual lesions while the body rebuilds systemic immune system during the post-transplant period, providing an excellent immune microenvironment for the vaccination. Lacy et al. reported a phase II trial through which a comparison was conducted between 27 patients who treated with Id-DC vaccine after auto-HSCT and 124 patients who only received auto-HSCT during the same period. Although a discrepancy in PFS was not observed, a statistically and clinically significant improvement in overall survival(OS) was observed (94).

However, DC-based vaccines in populations with advanced disease progression and post-HSCT could not be successfully translated into clinical efficacy probably because of a higher tumor burden and/or immune compromise due to strong chemotherapy treatment (95). Röllig et al. used mature monocyte-derived Id-pulsed DCs and KLH to explore the potential of DC vaccines in stage I myeloma (96). Their results suggest that DC vaccine is a viable approach to elicit T-cell immune responses in patients with early stage myeloma.

#### 4.2.2 MM-associated antigen peptide/mRNA

The selection of appropriate tumor-associated antigens (TAAs) is critical for developing a vaccine therapy to maintain tumor specificity and immunologic efficacy. MUC1 is TAA expressed in all MM cells and in the serum of MM patients. ImMucin is a 21mer synthetic long peptide vaccine that encodes the signal peptide domain of the MUC1, possessing high density of T and B cell epitopes, and is able to elicit strong MUC1-specific T and B cell responses (97). New York esophageal squamous cell carcinoma 1(NY-ESO-1) is considered to be the most immunogenic cancer testicular antigen (CTA). In patients with advanced MM, NY-ESO-1 expression mediates spontaneous humoral and CD8+ T cell immunity (98). In contrast, PTD-NY-ESO-1 moDCs by proteins transduced appears to induce stronger CD8+ immune responses (99).

Researchers reported a CTA-mRNA-loaded vaccination for the treatment of MM. They extracted and prepared autologous moDCs pulsing KLH, and electroporated with mRNA for the melanoma antigen family A, 3 (MAGE-A3), SURVIVIN, and BCMA. This study showed that TAA-mRNA of mature electroporated DCs could induce TAA-T cell immune responses in myeloma patients after HSCT (100). CT7 and MAGE-A3 are the most common CTA in MM and WT1 is expressed in the bone marrow. They may be associated with high tumor load and disease recurrence. The Phase 1 clinical trial carried out by Chung et al. demonstrated the feasibility of preparing a DC vaccine from autologous Langerhans-type dendritic cells (LC) electroporated by CT7, MAGE-A3 and WT1 mRNA. It was observed that CD34+ haematopoietic progenitor cells (HPC)-derived LC could be more effective in activating cytotoxic T lymphocytes (CTL) to mount specific anti-myeloma immune responses (101).

#### 4.2.3 Whole MM antigen-loaded DC

Single-antigen DC vaccines have a potential limitation in that their antitumor effects can be affected by immune evasion if downregulation of antigen expression occurs. In myeloma, an alternative to target Id or TAA is the use of total MM-antigen spectrum loaded ex-vivo-generated moDCs. This approach makes it possible for multivalent immune responses to target tumor-specific neoantigens. Whole MM antigen-loaded DC technology for vaccine preparation includes the establishment of DC-tumor fusion cells, pulsing DCs with myeloma lysates, pulsing DCs with myeloma apoptotic bodies and loading of DCs with tumor exosomes or whole cell DNA or RNA (90).

Rosenblatt et al. reported outcomes in Phase II clinical trials in which DC-tumor fusion cells vaccines were used to target MRD in myeloma patients after HSCT. Although the treatment with DC-vaccines after HSCT promoted the tumor-specific T cells proliferate, no significant differences in T-cell responses were seen in patients who received vaccine therapy before transplantation from those who only received vaccine therapy after transplantation (102). The efficacy of vaccination using DCs pulsed with Id and tumor lysate were assessed in myeloma mouse model by Hong et al. They found that tumor lysate-pulsed DCs vaccines were more efficient in protecting mice against developing myeloma, delaying the progression of tumor, and inducing tumor regression against established tumor, suggesting a more pronounced advantage of myeloma cells themselves as a source of tumor antigens over Id proteins (103). Vasileiou et al. designed a preclinical study to explore alternative whole-tumor antigen approaches, i.e. phagocytosis of apoptotic bodies of autologous myeloma cells or total RNA transfection by electroporation. Both approaches are effective in inducing specific CD8 CTL and deserve further investigation in clinical trials (104). **Table 1** summarizes the key clinical trials.

**Table 1:** MM vaccine clinical trials.

Numbers of participants	Stage of disease	DC type	Tumor antigen	Antigen Loading/Adjuvant	Key results	Reference
5	stage IIA IgG myeloma	/	Id	GM-CSF	Id-specific T-cell responses	85
6	stage I IgG myeloma	/	Id	IL-12 alone or combined with GM-CSF	4/6 reduction of MM; 3/4 Id-specific T-cell responses 2/6 unchanged level of blood tumor cells; 1/2 mounted T-cell response	86
15	advanced myeloma	/	Id	Chemically linked Id-phage GM-CSF as adjuvant and KLH as control antigen	11/15 reduction or stabilization of paraprotein levels and/or 24-hour light chain excretion; 4/5 anti-Id humoral response; 15/15 demonstrated high levels of specific antibodies; 14/14 cellular immune response	88
27 in Vaccine group 124 in database	8/27 and 33/124 in stage II 19/27 and 91/124 in stage III	APC8020 (Myloven)	Id	DC precursors were co-cultured with patient’s serum as a source for Id	trial patients:6/26 CR; 2/26 PR; 19/27 SD trial group vs database group: TTP 1.5 years vs 1.6 years median PFS no statistically significant difference median OS 5.3 years vs 3.4 years	94
9	stage I	Mo-DC	Id	Mature monocyte-derived Id-pulsed DCs and KLH	5/9 Id-specific T cell proliferation; 8/9 Id-specific cytokines produce; 3/9 decrease in M protein; 5/9 stable M protein	96
15	ISS score: 5/15 I, 7/15 of II, 3/15 of III	/	MUC1	ImMucin vaccines co-administered with GM-CSF	15/15 specific T cell response; 10/15 anti-ImMucin IgG antibody response; 9/10 significantly reduce the levels of soluble MUC1	97
12	stage II and III	moDC	MAGE3 SURVIVN BCMA	autologous moDCs pulsing KLH, and electroporated with mRNA	12/12 anti-KLH T-cell responses; No KLH antibodies	100
10 in vaccine treatment arm 10 in control arm	9/10 VGPR 1/10 CR (both arms)	LC	CT7 MAGE-A3 WT1	CD34+ HPC-derived LCs electroporated with mRNA	(vaccine treatment arm) 3 months post-ASCT: 3/10 MRD negative CR; 3/10 MRD-positive CR 4/10 VGPR; 12 months post-ASCT: 5/10 MRD-negative CR;3/10 MRD-positive CR 1/10 VGPR 1/10 relapsed disease;	101
Group1:24/26 were vaccinated Group2:12/9 were vaccinated	No clinical stage specified	moDC	MM cells	moDCs cultured with GM-CSF, IL-4 and TNF-α fused with autologous bone marrow-derived MM cells	Vaccination resulted in all evaluable patients demonstrated at least a twofold expansion of myeloma specific CD4+ and/or CD8+ T cells.	102

In MM, the combination of vaccines with existing treatment modalities is thought to play a synergistic role in improving the strength and duration of the immune response (105). We have explored some examples of vaccines combined with HSCT therapy above. The post-transplant status of MM patients provides a window for vaccine-stimulated anti-myeloma responses and targeting of MRD (102). Previous studies have shown that lenalidomide enhances vaccine anti-tumor responses and vaccine-specific cellular and humoral immunity, potentially as an adjuvant for cancer vaccines (106). In the mouse model, lenalidomide synergistically augments the efficacy of the DC vaccine by inhibiting the production of immunosuppressive cells, effectively inducing Th1-specific immune responses and reducing Th2-specific immune responses. In addition, lenalidomide enhances the activation and proliferation of NK cells in mice, obtaining higher levels of NK cell-mediated cytotoxicity in conjunction with the vaccine (107). Immune checkpoint PD-1/PD-L1 is an essential pathway for tumor-mediated immunosuppression. DC/myeloma cell fusion vaccines were investigated for high expression of PD-L1, which may provide inhibitory signaling and weaken vaccine immunity. In contrast, blocking PD-1 promotes vaccine-induced Th1 cell polarization, reverses the upregulation of PD-1 expression, reduces Treg cells and enhances anti-tumor immunity (108). Furthermore, the triple therapy of DC vaccine, PD-1 blockade and lenalidomide in the MM mouse model potently inhibited the growth of tumor cells, with enhancing functional activity of cytotoxic T lymphocytes and NK cells, synergistically enhancing anti-tumor immunity (109). The same results were observed in MM mouse models cotreatment with a triple combination of DC vaccine, PD-L1 blockade and pomalidomide (110).

In conclusion, from first- to second-generation vaccines, more and more vaccine preparation methods have emerged and strategies for recognizing tumor antigens have also been continually refined (90). However, it is difficult to standardize the vaccine preparation as well as the timing and route of vaccine use, due to the small number of patients included in the trials (111). With the complex immune microenvironment in MM patients, certain immune dysregulation and deficiencies in the quantity and function of immune cells affect the efficacy of vaccination. Based on the current use of vaccines in MM, we need to further refine our vaccine strategy, explore the combination with vaccines and other agents and achieve specialization and standardization of vaccine preparation to make it an effective clinical treatment.

### 4.3 Enhancing antigenicity

MM is oncogene-dependent. Cytogenetics suggests that MM has a diverse genome, highlighted by structural rearrangements and copy number exceptions, and these exceptions determine the progression and final outcome of subsequent disease. Oncogenic mutations are more clonal while disordered driver genes represent a worse prognosis (112). Mutations in the coding region caused by genetic instability in carcinogenesis can cause amino acid sequence changes. New proteins, known as tumor antigens, can activate the body’s immune system and elicit effect immune responses (113). Increased expression or presentation of tumor antigens enhance tumor cells antigenicity. Based on their characteristics, tumor antigens are generally classified into three broad categories, i.e., tumor-associated antigens (TAAs), cancer testicular antigen (CTA) and tumor specific antigens (TSA). Most TAAs are embryonic antigens and might have induced immunological tolerance, making it difficult to develop a specific and durable immune response. Another risk of using TAAs as the immune target is the induction of autoimmunity against the corresponding normal tissues (114).

TSA, also known as neoantigen, is another effective target for tumor cell immunotherapy that has emerged in recent years. How neoantigen come to defined is still not well understood so far. Some researchers believe that these tumor neoantigens are generated by non-synonymous or other genetic changes (115). Another popular theory is that neoantigen come from peptides of tumor proteins with altered characteristics (116).

Somatic mutations caused by any mechanisms could generate tumor neoantigens. The RNA sequences from the MM Research Foundation CoMMpass Study were used for the identification of Intron retention (IR) events and the prediction of IR-neoantigens. It was found that high IR-neoantigens load was related to poor overall survival and unfavorable clinical outcome (117).

Wells et al. believe that reliable neoantigen predictions is dependent on the understanding of key parameters governing the immunogenic epitope (118). The prediction of neoantigen can be accomplished by using bioinformatics algorithms. Massively parallel sequencing (MPS) can identify specific somatic mutations by comparing DNA sequences of tumor cell and normal host cell origin. This process usually selects exon sequencing, in order to significantly reduce costs and complexity of analysis (114). However, due to human leukocyte antigen(HLA) restriction, not all mutations result in new epitopes that can be recognized by the immune system. Computer analysis is used to predict the affinity of new epitopes for HLA binding and to screen for neoantigens that most likely to induce potent T-cell immune response. Current predictions of antigenic epitopes focus on MHC I-binding epitopes, and the identification of major histocompatibility complex(MHC) II epitopes is complicated by the characteristics of MHC II peptide binding (119). From the outcomes of somatic mutations and gene-expression profiling, Jian et al. developed a neoantigen-prediction pipeline and constructed a neoantigen immune response score. This approach can be used to rapidly identify the new antigens created from somatic mutations and predict OS (120).

It is generally accepted that high somatic mutation rates are associated with increased genomic instability and reduced overall survival (121). The mutational load associated with MM is lower compared with other types of cancer. In a study involving 663 MM patients, a method combining exome sequencing and HLA binding prediction was used to determine mutational load and predict neoantigen load in MM patients. The mutational load was found to be directly proportional to the predicted neoantigen load. Survival analysis showed that PFS for the patients with higher somatic missense mutational load and predicted neoantigen load than mean values was significantly shortened and it was independent of disease stage and cytogenetic genetic abnormalities (122).

Permual et al. reported neoantigen-specific T cell responses in MM patients. This is the first study that experimentally validates the ability of triggering an immune response by neoantigens predicted from the next generation sequencing (NGS) in relapsed MM patients. Their data support that neoantigens are able to elicit cytotoxic CD8+ T cell activity in the setting of combination immunotherapy (123). Hence, neoantigens can form complex with human leucocyte antigen (HLA) molecules and presented to T cells, where the complex is recognized by TCR as “non-self”. Anti-tumor specific response is induced and not affected by central and peripheral immune tolerance and not causing damages to normal tissues. For this reason, tumor neoantigens are excellent targets for immunotherapy.

Studies have revealed that tumor-infiltrating lymphocytes(TILs) show strong neoantigen-killing ability once ICIs block the inhibitory signals from tumor cells, boosting the efficacy of ICIs (124). Strategies of combining targeted neoantigens can reduce relapse and side effects in patients undergoing bone marrow transplantation. Foglietta et al. immunized donors with recipient-derived Id, conjugated with KLH and observed anti-KLH and anti-Id cellular immune responses. Similarly, anti-neoantigen immune responses were detected in recipients after HSCT. These observations prove that neoantigen and tumor antigen-specific humoral and cellular immunity can be safely induced in HSCT donors and passively transferred to recipients (125).

It is well understood that tumor neoantigens are abundantly expressed in tumor cells, rarely expressed in normal somatic cells and are not subjected to thymic negative selection. Tumor antigens are natural targets for vaccine preparation. Bekri S. et al. experimentally proved that tumor antigen-specific CD4 (helper) T cells can provide protective antitumor immunity and against antigens that are not expressed by the vaccine through activating CD8 T cells (126). This may be associated with enhanced antigen cross-presentation by CD4+T, production of cytokines and upregulation of MHC II molecule protein (119).

Immunotherapy targeting neoantigens is an emerging strategy for personalized immunotherapy and has resulted in prolonged PFS and OS in clinical trials. However, these approaches still face a huge set of challenges such as the collection of more data on neoantigen mutations, the improvement of neoantigen prediction algorithms, and the exploration of optimal neoantigen targets. Studies on the combination of neoantigen-targeted therapies and other immunotherapies are being carried out and will definitely benefit more patients.

### 4.4 Increasing the expression of co-stimulatory molecules

Sensitizes tumor cells to immune attack by enhancing the propensity of immune effector cells to recognize and kill tumor antigens. The main approach mentioned here is to enhance the expression of signals on the surface of tumor cells. The signaling molecules on the surface of tumor cells and their interaction with T cells determine whether T cells can be successfully activated to produce anti-tumor immunity.

The desire to generate effective anti-tumor immunity requires APCs present and process antigens to activate T cells, and differentiated antigen-specific CD8+ T cells migrate and persist in the tumor environment. APCs present antigens via the classical MHC class I and class II pathways. Besides, there is a cross-presentation pathway, which refers to the presentation of exogenous antigens on MHC I and is essential in anti-tumor responses and antigen tolerance (127).

In general, T cell activation requires three signals. TCR-CD3 + MHC I/II +CD8/CD4 is the first signal for T-cell activation. T cells interact with co-stimulatory molecules on the surface of the APC to generate a second signal (co-stimulatory signal) for activating various types of T cell responses. CD28 is a protein expressed as a homodimer on T cell and is the main family of co-stimulatory molecules, with receptor B7-1/2 (CD80/86) on APC (9). Besides, the cytokines produced during T cell activation act as a third signal to further promote T cell proliferation and differentiation and to generate immune memory, while IL-2 plays a key role in this process.

MM cells are generally considered to be weak APCs and express activating co-stimulatory signals on their surface, such as CD40,CD80/86,4-1BBL,OX40L,CD70,B7 relative protein1(B7RP1) molecules that binding to CD40L,CD28,4-1BB,OX40,CD27, inducible co-stimulator(ICOS) receptors on T cells, respectively (9). The inability of general tumor antigens to activate T cells are associated with low expression of activating co-stimulatory molecules (21, 128). Enhancing the expression of these signals and subsequently strengthening susceptibility to immune attacks appears to be a potential therapeutic approach.

DCs are thought to play a dual role and except uptaking and processing myeloma antigens and activating specific CD8+ T cells, DCs can also bind to CD28 molecules on the surface of non-apoptotic myeloma plasma cells through CD80/86 molecules, allowing myeloma plasma cells to evade killing by CD8+ T cells (129). Nair et al. demonstrated that CD28 expression may be associated with myeloma cell survival, induces IL-6 and IDO production upon binding to CD80/86 ligands, and is a target for myeloma therapy (130).

Another class of co-stimulatory molecules is the TNF/TNF receptor family. OX-40 ligand binds to the T cell surface receptor OX-40, leading to the expansion of CD4+ T cells (131). 4-1BBL binds to the T cell receptor not only induces the T cell activation, but also prevents apoptosis of activated T cell, enhancing the anti-tumor effect (132). MM cells express only the weaker 4-1BBL and B7 molecules on their surface. Transduction of B7-1 and 4-1BBL in MM cell lines makes it possible to activate and amplify T cell and stimulate anti-myeloma immune response (133). Given the ability of lysing viruses to enhance tumor cells immunogenicity and induce anti-tumor immune responses, Wenthe et al. infected myeloma cells with immunostimulatory Lokon lysing adenovirus (LOAd) carrying trimeric membrane-bound CD40L (LOAd700, LOAd703) and 4-1BBL (LOAd703).Their results showed that T cells are successfully activated (134).

CD40 is expressed on the surface of myeloma cells instead of on normal mature plasma cells. The interaction of CD40 with CD40L mediates the mechanism of angiogenesis in MM patients (135). CD40-CD40L pathway restores sensitivity to immune attack in malignant tumor cells associated with activation of the Nuclear factor kappa-light-chain-enhancer of activated B cells (NF-κB)-mediated signalling pathway (136). Thus, the CD40-CD40L pathway can effectively promote systemic anti-tumor immune responses, while targeting CD40 can directly inhibit tumor proliferation, and metastasis, and enhance tumor cells susceptibility. Advances in clinical trials and treatment of the CD40-CD40L pathway as an immune checkpoint have been discussed in detail in the review by Tang et al, and treatment targeting CD40/CD40L in MM still needs to be further explored (137).

## 5 Conclusion

Researching into the molecular mechanisms of immunodeficiency in MM provides more options for myeloma patients who are resistant to classical treatment. Nevertheless, due to the disease characteristics of myeloma itself, genomic and clonal evolution in the bone marrow immune microenvironment and the immune deficiency leading to eventual disease relapse, new treatment strategies are still imminent (138). As the continuous exploration of immunotherapy approaches, patients have led to prolonged PFS and overall remission rates. However, the problem of disease recurrence and treatment resistance still exists. Here we discuss the activation of specific anti-tumor immune responses mediated by targeting MM cells as a potential solution strategy to overcome the diminished effect of immunotherapy due to antigen loss. The therapeutic modality of targeting tumor cells is achieved by enhancing auto-immunogenicity of MM cells, increasing the expression of neoantigens and increasing the expression of co-stimulatory molecules. Some of these treatment strategies are still in the pre-clinical stage and lack large-scale clinical trials to prove that they can benefit the majority of patients.

In conclusion, we are summarized the therapeutic strategies to enhance the immune response based on multiple myeloma cells and these treatments have shown excellent clinical efficacy and deserve further exploration.

## Author contributions

ZL: Conceptualization, Writing - Review and Editing, Project administration. CY: Writing - Original Draft, Visualization, XL: Resources. XX: Resources. XZ: Resources. RF: Supervision, Funding acquisition, Project administration. All authors contributed to the article and approved the submitted version.
